# *HIC1* (Hypermethylated in Cancer 1) modulates the contractile activity of prostate stromal fibroblasts and directly regulates *CXCL12* expression

**DOI:** 10.18632/oncotarget.27786

**Published:** 2020-11-10

**Authors:** Marion Dubuissez, Sonia Paget, Souhila Abdelfettah, Nathalie Spruyt, Vanessa Dehennaut, Gaylor Boulay, Ingrid Loison, Clementine de Schutter, Brian R. Rood, Martine Duterque-Coquillaud, Xavier Leroy, Dominique Leprince

**Affiliations:** ^1^University Lille, CNRS, INSERM, Institut Pasteur de Lille, UMR9020-UMR-S1277 - Canther - Cancer Heterogeneity, Plasticity and Resistance to Therapies, Lille, France; ^2^Department of Pathology and Center for Cancer Research, Massachusetts General Hospital and Harvard Medical School, Boston, MA, USA; ^3^Center for Cancer and Immunology Research, Children's National Medical Center, Washington, DC, USA; ^4^Department of Pathology, University Lille, Lille, France; ^5^Present Address: Maisonneuve-Rosemont Hospital Research Center, Maisonneuve-Rosemont Hospital, Montreal, Canada; ^*^These authors contributed equally to this work

**Keywords:** HIC1, CXCL12, SDF1, α-SMA, Acta2

## Abstract

*HIC1* (*Hypermethylated In Cancer 1*) a tumor suppressor gene located at 17p13.3, is frequently deleted or epigenetically silenced in many human tumors. *HIC1* encodes a transcriptional repressor involved in various aspects of the DNA damage response and in complex regulatory loops with P53 and SIRT1. *HIC1* expression in normal prostate tissues has not yet been investigated in detail. Here, we demonstrated by immunohistochemistry that detectable *HIC1* expression is restricted to the stroma of both normal and tumor prostate tissues. By RT-qPCR, we showed that *HIC1* is poorly expressed in all tested prostate epithelial lineage cell types: primary (PrEC), immortalized (RWPE1) or transformed androgen-dependent (LnCAP) or androgen-independent (PC3 and DU145) prostate epithelial cells. By contrast, *HIC1* is strongly expressed in primary PrSMC and immortalized (WMPY-1) prostate myofibroblastic cells. *HIC1* depletion in WPMY-1 cells induced decreases in α-SMA expression and contractile capability. In addition to *SLUG*, we identified stromal cell-derived factor 1/C-X-C motif chemokine 12 (*SDF1/*CXCL12) as a new HIC1 direct target-gene. Thus, our results identify *HIC1* as a tumor suppressor gene which is poorly expressed in the epithelial cells targeted by the tumorigenic process. *HIC1* is expressed in stromal myofibroblasts and regulates *CXCL12/SDF1* expression, thereby highlighting a complex interplay mediating the tumor promoting activity of the tumor microenvironment. Our studies provide new insights into the role of HIC1 in normal prostatic epithelial-stromal interactions through direct repression of *CXCL12* and new mechanistic clues on how its loss of function through promoter hypermethylation during aging could contribute to prostatic tumors.

## INTRODUCTION

Prostate cancer (PCa) is a frequently diagnosed cancer in western countries and is still the second leading cause of death in men [1]. A distinguishing feature of prostate cancer is that its incidence increases with age [2]. Indeed, PCa arise from preneoplastic prostate intraepithelial neoplasia (PIN) commonly found in men by their fifties, which progress into androgen-dependent localized cancers in men around 60–70 years old. Whereas these adenocarcinomas initially respond well to androgen-deprivation therapy (ADT), most patients relapse and progress to androgen-independent more aggressive and metastatic forms of prostate cancer with poor prognosis. Numerous studies strongly suggest that stromal cells are of paramount importance in prostate cancer. In the tumor microenvironment, reciprocal interactions between stromal and epithelial cells are implicated in tumor initiation, growth and metastasis. In the tumor-associated stroma, the secretion of several cytokines is increased, thereby contributing to the emergence of Cancer-Associated Fibroblasts (CAFs). In addition, stromal cell-derived factor (SDF-1) also known as C-X-C motif chemokine 12 (CXCL12), secreted by stromal fibroblasts binds its cognate receptor CXCR4 on epithelial cells to trigger several downstream pathways involved in cell proliferation, cancer cell invasion and tumor angiogenesis [3–5].

**Figure 1:**
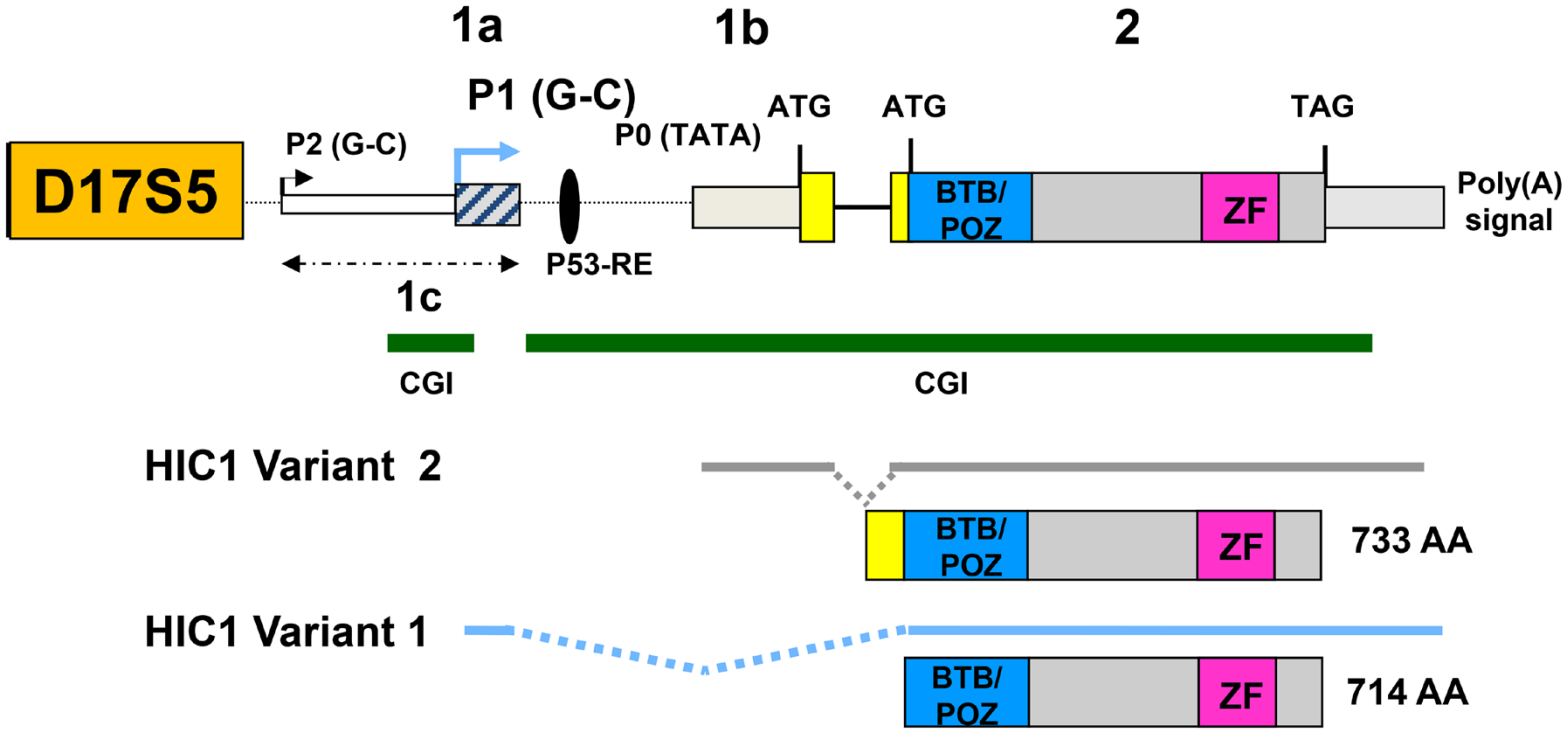
Genomic organization of the human *HIC1* locus. The structure of the human *HIC1* locus with a large coding exon (exon 2) and alternate 5′ exons as derived from several studies is schematically drawn [6, 22, 23]. The two major promoters called P1 and P0 as well as the minor P2 promoter generating *HIC1* transcripts with heterogeneous 5′ ends are shown. For clarity, only the two major transcripts generated by alternative splicing, variant 1 (1a-containing, driven by a GC-rich promoter, NM_006497) and Variant 2 (1b-containing, driven by a TATA-box promoter NM_001098202) have been shown below the human *HIC1* genomic locus [23]. The variant 1 transcripts are by far the most abundant *HIC1* transcripts [22, 23]. A similar organization is found in mice [21, 22]. Two conserved CpG islands (CGI), shores and shelves identified in the human and mouse *HIC1* locus are shown as green lines [10, 35].

*HIC1* (Hypermethylated in Cancer 1) is a tumor suppressor gene located at 17p13.3 on the short arm of chromosome 17 [6] (Figure 1), a region frequently silenced by hypermethylation or deleted by loss of heterozygosity (LOH) in many human cancers including breast [7, 8], colon [6, 9, 10], lung [11] and prostate carcinomas [12–15], particularly in metastatic PCa [16]. Expression of *HIC1* is associated with an improved prognosis in human breast [8] and lung [11] carcinomas. Surprisingly, in colorectal carcinomas, high *HIC1* expression is correlated with decreased survival despite a better response to chemotherapy [10]. *HIC1* is also hemi-methylated in normal breast tissue [7], cerebellum [17] and normal prostate as well as in benign hypertrophic tissue (BHP) [12]. *Hic1* heterozygous mice (*Hic1+/*−) develop a spontaneous gender-and age-dependent spectrum of tumors associated with silencing of the remaining wild-type allele by promoter hypermethylation [18]. As a whole, these data demonstrate that *HIC1* silencing through epigenetic mechanisms predispose many tissues to tumorigenesis.

*HIC1* encodes a transcriptional repressor containing an N-terminal BTB/POZ domain and five C-terminal C_2_H_2_ *Krüppel*-like Zinc fingers able to bind to a specific motif called HiRE (HIC1-Responsive Element) centered on a GGCA core motif [19, 20].

Here, we have analyzed the expression of HIC1 in normal prostate tissues by immunohistochemistry. Strikingly, HIC1 protein expression was not detected in glandular epithelial cells but rather in the nuclei of dispersed stromal cells. Similar results were obtained in prostate adenocarcinomas. RT-qPCR analyses on a cohort of 30 prostate tumor samples of different grades demonstrated that high *HIC1* expression is found in stroma-rich prostate adenocarcinoma. In addition, *HIC1* expression was barely detectable by RT-qPCR analyses in transformed androgen-dependent LnCAP or androgen-independent (PC3 and DU145), immortalized (RWPE1) or normal primary (PrEC) human prostate epithelial cells. By contrast, *HIC1* expression was detected in primary human smooth muscle cells from prostate stroma (PrSMC) and in the immortalized prostate stromal myofibroblastic cell line WPMY-1. α-SMA expression was reduced upon *HIC1* depletion in WPMY-1 cells, which resulted in a decrease of their contractile capability. Furthermore, we demonstrate that HIC1 directly regulates *CXCL12/SDF1* expression in WPMY-1 stromal myofibroblasts and in normal BJ-hTERT human fibroblasts as shown by siRNA interference and by chromatin immunoprecipitation (ChIP) of endogenous HIC1.

## RESULTS

### Immunohistochemical assessment of *HIC1* expression in normal prostate and in prostate adenocarcinomas

**Figure 2:**
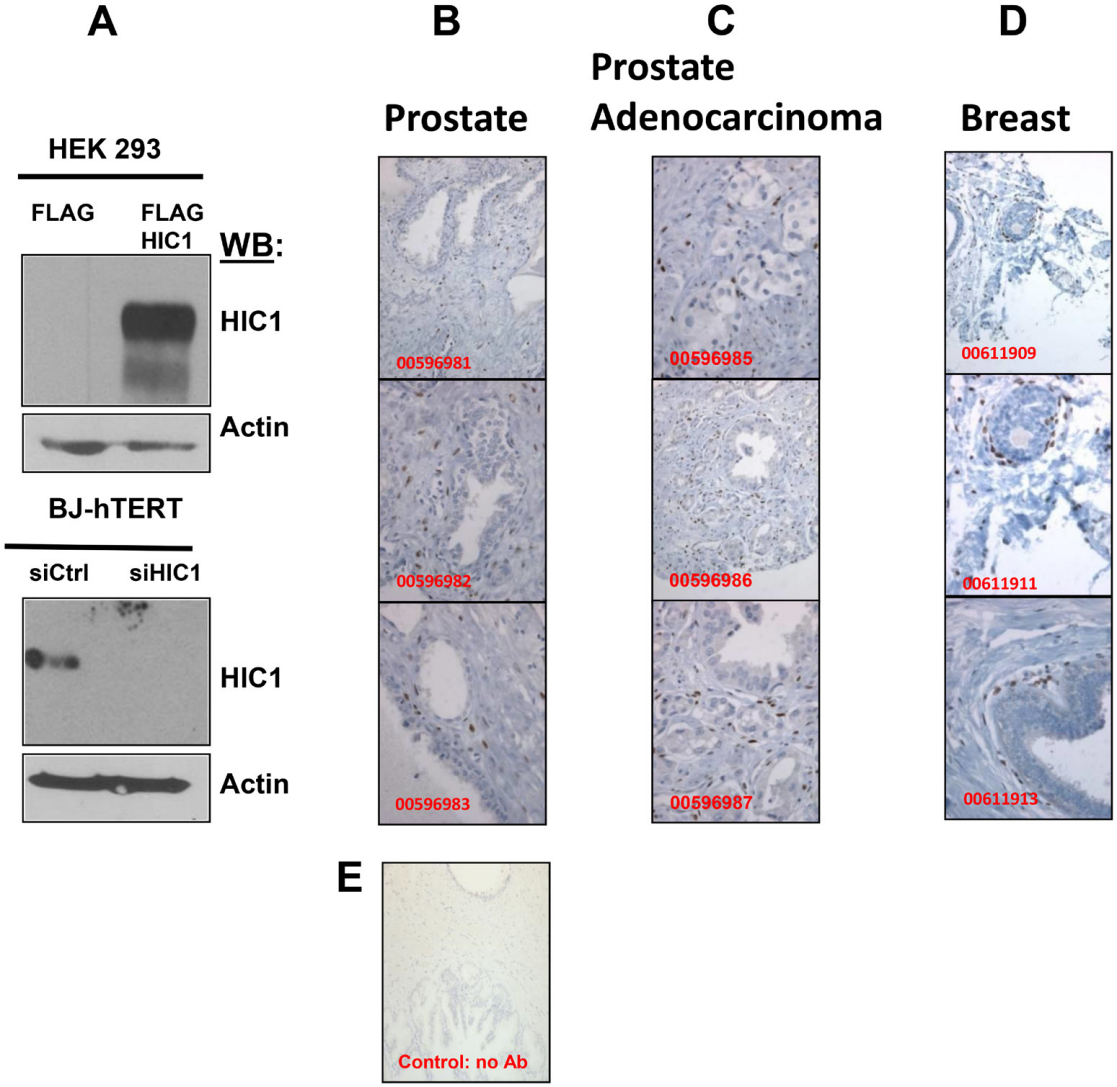
Immunohistochemical analyses of HIC1 expression in normal human prostate and breast tissues as well as in human PCa tissues. (**A**) Validation by Western blot analyses of the HIC1 antibody used in the immunohistochemical analyses. *Top panel*. HEK293T cells were transfected with the indicated expression vectors. After 48 hours, whole cells extracts were prepared and immunoblotted with the HIC1 antibodies (anti-HIC1 abcam 33029; dilution 1/1000) and actin as loading controls. *Bottom panel*. A similar experiment was performed with extracts of normal immortalized human BJ-hTERT fibroblasts transfected with control siRNAs (siCtrl) or a pool of siRNAs (Dharmacon) targeting *HIC1* (siHIC1) [25] to validate the efficient and accurate detection of endogenous HIC1 proteins by this antibody. (**B**–**D**) Immunohistochemical analyses. Tissue sections from normal prostate (B), from prostate adenocarcinomas (C) and from normal breast (D) stained by immunohistochemistry using the anti-HIC1 antibodies. The sections were counterstained by hematoxylin (blue). The original magnification is ×100 for all panels except for panels 00596985, 00596987, 00611913: ×200. (**E**) As a negative control for immunostaining, a tissue section from normal prostate was stained by immunochemistry as in panels (B–D) without any primary HIC1 antibody. (magnification: ×200).

We have analyzed by immunohistochemistry the expression of HIC1 in normal prostate tissues using an affinity purified anti-human HIC1 antibody recommended for IHC (Abcam, ab33029). As control of its specificity, we demonstrated through Western blots analyses that this antibody detected HIC1 overexpressed in HEK293T cells and the endogenous HIC1 proteins in immortalized human fibroblasts BJ-hTERT but not in BJ-hTERT knocked-down for *HIC1* (Figure 2A). Surprisingly however, in IHC experiments using this antibody, HIC1 protein expression was not detected in normal epithelial cells but rather in disseminated cells in the prostate stroma as a strong nuclear staining in agreement with its function as transcription factor (Figure 2B). In several prostate adenocarcinomas, this ab33029 HIC1 antibody failed to detect any significant HIC1 expression in cancer cells but again a predominant expression in the stromal compartment (Figure 2C). Our observations were reproducible in normal breast tissues where we also detected by imunohistochemistry HIC1 expression in the stroma and in cells adjacent to the ducts but not in the epithelial cells of the ducts (Figure 2D). As negative control, no immunohistochemical staining of normal prostate tissue was observed in the absence of primary HIC1 antibodies (Figure 2E).

As a whole, these experiments demonstrated that HIC1 is not detectable by immunohistochemistry in normal or transformed prostate epithelial cells but is expressed in normal and tumor stroma.

### *HIC1* expression in human primary prostate tumors is correlated with stromal content

**Figure 3:**
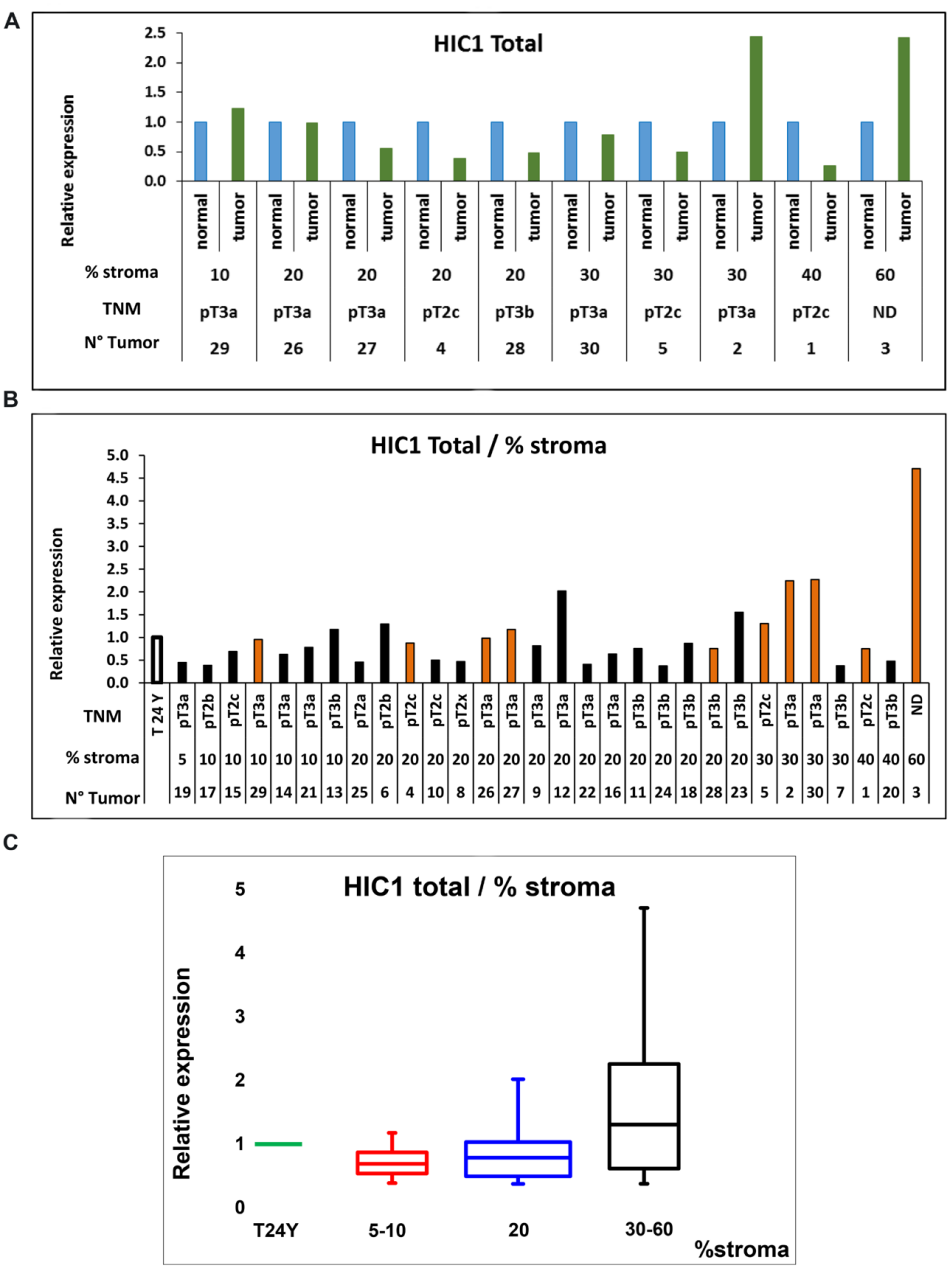
RT-qPCR analyses of *HIC1* expression in normal human prostate and human PCa tissues. (**A**) Profiles of *HIC1* expression in 10 pairs of cancerous and matched normal prostate tissues. Total RNAs were extracted and quantitative expression of the two major *HIC1* isoforms’ expression was measured by RT-qPCR analyses. Results are normalized to *ALAS1* RNA [51] or *18S* rRNA and are presented as means +/− S.D. from two experiments in triplicate. For each patient, the percentage of normal stroma present in the tumor samples which was used as the ranking criteria is indicated below as well as the TNM staging (see Supplementary Table 1 for details). (**B**) Analyses of *HIC1* expression in prostate tumors. A cohort of 20 tumors for which the matched normal tissue was not available was analyzed by RT-qPCR analyses (black column) as described above except that the control corresponded to RNAs extracted from the prostate of a healthy young (24 year-old) donor (Biochain) as control. The 10 tumors used in panel A were also included in this study (orange column). (**C**) Quantification of *HIC1* expression in the 30 prostate tumors clustered in subgroups according to their stromal content. *HIC1* expression measured by RT-qPCR analyses of the 30 tumors in panel B is represented as box plots in comparison with its expression in a normal prostate tissue obtained from a young 24-years old donor. The box area corresponds to the first and third quartile. The median is shown as a horizontal line in the box. The maximum and minimum values are indicated by the whiskers above and below the box.

To consolidate these results, human primary prostate cancers (PCa) samples were assessed for *HIC1* expression using RT-qPCR of total isolated RNAs (Supplementary Table 1). For these experiments, we used a pair of oligonucleotides located in the large *HIC1* coding exon 2 in order to amplify the two major alternative *HIC1* transcripts, *HIC1 1a* (variant 1) and *HIC1 1b* (variant 2) (Figure 1) [21–23]. We first determined *HIC1* expression levels in 10 pairs of prostate cancer tissues and matched adjacent non-cancerous tissues. We found that *HIC1* was expressed in all tumors in agreement with our IHC results (Figure 3A). In most cases (6 out of 10), *HIC1* was expressed at lower levels than in the adjacent normal tissues as shown in numerous studies addressing various types of solid tumors. However, *HIC1* was detected at higher levels in cancerous tissues than in normal tissue in two tumors enriched in stroma, as already observed in some colorectal carcinomas [10].

In a second experiment, we included these 10 tumors in a larger cohort with 20 prostate cancer samples (Supplementary Table 1) and we measured *HIC1* expression levels by RT-qPCR using the normal prostate of a young (24-year old) healthy donor as control [24]. *HIC1* expression was detectable in all samples. Strikingly, high expression levels were observed in some of the most advanced tumors, pT3a and pT3b according to the TNM classification for prostate cancer and again particularly in tumors with high stromal content (Figure 3B and 3C).

Thus, *HIC1* expression is detectable in prostate tumor tissues and a trend is observed with the amount of stroma present in our cohort of samples.

### *HIC1* is detected in primary stromal smooth muscle cells (PrSMC) but expressed at a much lower level in human primary epithelial prostate cells (PrEC)

**Figure 4:**
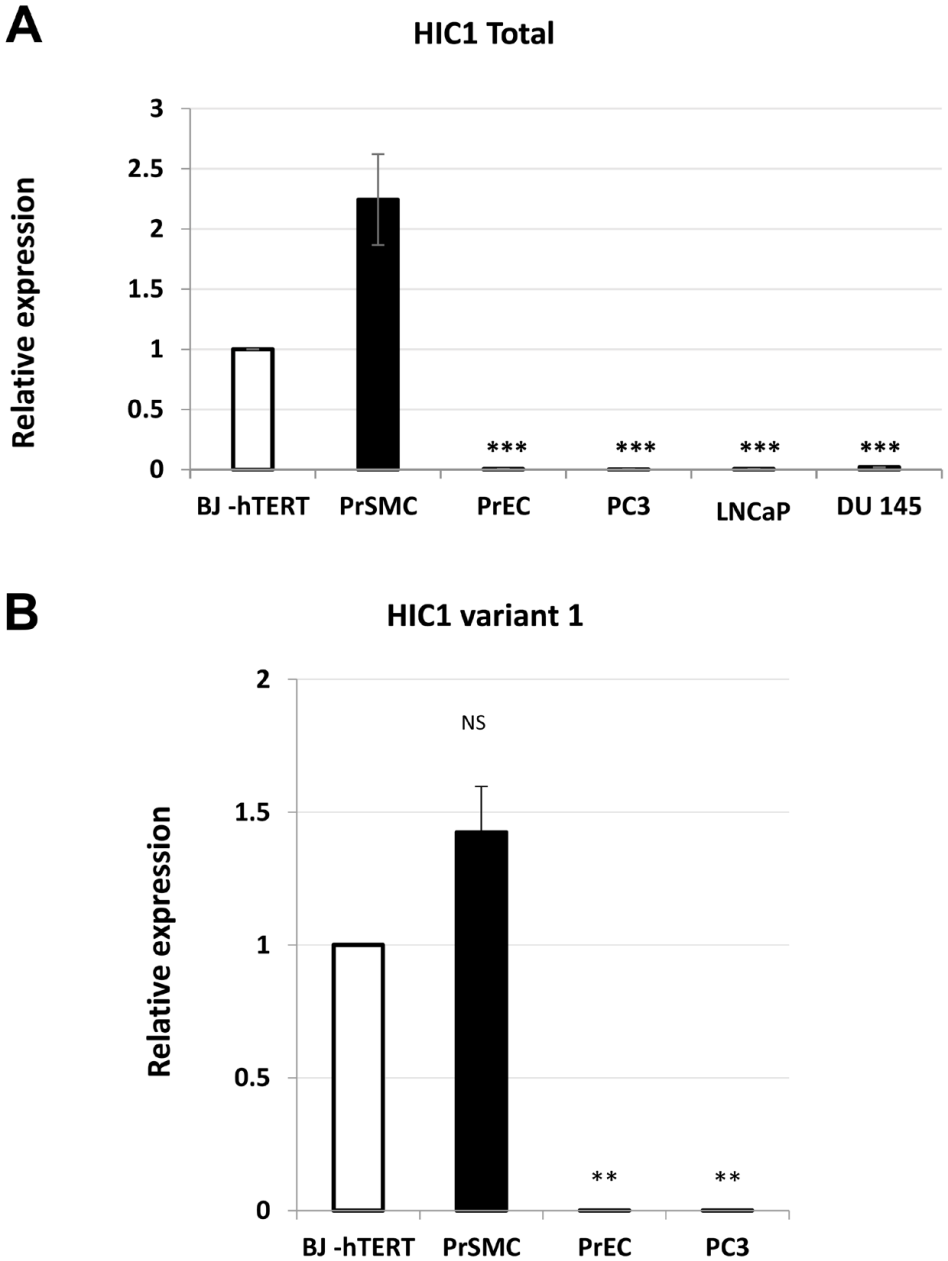
Analyses of *HIC1* expression in various human normal and transformed prostatic cell types. (**A**) Global expression level of *HIC1*. The global expression level of *HIC1* was examined in normal primary prostate epithelial cells (PrEC) and stromal smooth muscle cells (PrSMC) as well as in transformed androgen-dependent (LNCaP) and androgen-independent (DU145, PC3) prostate cancer cell lines by RT-qPCR analyses with two primers located in the large common coding exon, exon 2. A “no-RT” control was included in all experiments. (**B**) The major *HIC1* transcript, variant 1, is expressed in PrSMC. A similar experiment with some of the samples used in panel (A) was conducted with nested primers (sense primer in the alternative 1a exon and the antisense in the common exon 2) specifically amplifying the variant 1 transcript.

*HIC1* expression has already been described in many fibroblast cell lines including WI38 and IMR90 [6], MRC5 [22] and BJ-hTERT [25]. However, *HIC1* mRNAs levels in various types of primary prostate cells, including epithelial and stromal cells have not been investigated in detail. In the prostate stroma, the two predominant cell types are fibroblasts and smooth muscle cells also called myofibroblasts. Therefore, we measured *HIC1* mRNA expression levels by RT-qPCR in normal primary human prostate epithelial (PrEC) and smooth muscle (PrSMC) cells and in the transformed androgen-dependent LNCaP and androgen-independent PC3 and DU145 prostatic cell lines. Normal immortalized BJ-hTERT human fibroblasts were used as positive controls in our assays. *HIC1* was detected at high levels in both normal smooth muscle PrSMC cells and BJ-hTERT when compared to primary epithelial cells, PrEC (Figure 4A). In line with our observations at the protein level by IHC (Figure 2), *HIC1* expression was not detected in the three transformed epithelial cell lines LNCaP, PC3 and DU145, in close agreement with the silencing of *HIC1* through promoter hypermethylation in prostate tumors [12–16] (Figure 4A). To exclude contamination by genomic DNA, we reproduced these RT-qPCR analyses with nested oligonucleotides able to specifically amplify the major *HIC1* variant 1 transcript driven by the upstream G-C rich-promoter 1a (Figure 1). Again, significant expression of *HIC1* was detected in PrSMC and in BJ-hTERT fibroblasts together with very low levels in primary normal epithelial cells, PrEC and in transformed PC3 cells (Figure 4B).

**Figure 5:**
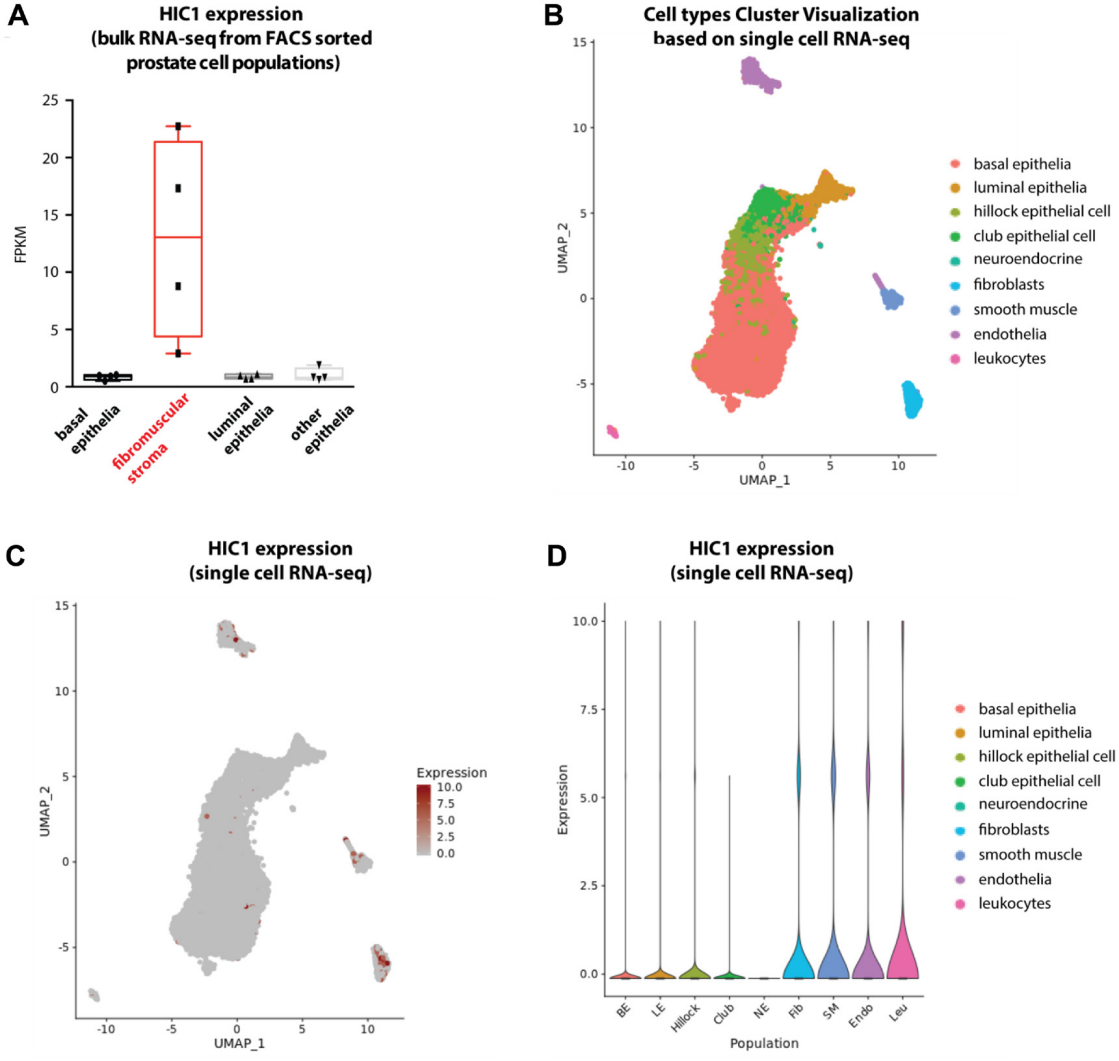
Analyses of *HIC1* expression in normal human prostate tissue by bulk RNA-sequencing and single cell RNA-sequencing. (**A**) Boxplot shows *HIC1* expression levels detected by bulk RNA-sequencing in FACS-sorted cell populations from normal human prostate tissues (GSE120716) [26]. *HIC1* was specifically detected in fibromuscular stroma. (**B**) Cell types cluster visualization based on single cell RNA-sequencing of normal prostate tissues. Cell types are color-coded and legend is found on the right. (**C**) UMAP plot shows *HIC1* expression levels as detected by single cell RNA-sequencing. (**D**) Violin plot shows *HIC1* expression levels as detected by single cell RNA-sequencing. Single cell RNA-sequencing of human prostate tissue was retrieved from Dr Douglas STRAND’s laboratory interactive website (https://www.strandlab.net/).

To further explore lineage specific expression of *HIC1* and validate our observations, we used a recently published dataset in normal human prostate tissues [26]. *HIC1* was specifically detected by bulk RNA-sequencing in a FACS-sorted fibromuscular stroma cell population when compared to epithelial cell populations (Figure 5A). In the same study, further investigations through single cell RNA-sequencing of dissociated normal prostate tissue allowed cell types cluster visualization (Figure 5B) and we clearly identified enrichment of *HIC1* expressing cells in the stromal compartment (Figure 5C and 5D, fibroblasts, smooth muscle, endothelia and leukocytes).

Thus, *HIC1* is only weakly expressed in normal human prostate epithelial cells, which are the final target of the transformation process, but is expressed in stromal cells which play a crucial role in tumor initiation and progression through cross-talk with epithelial cells.

### *HIC1* is strongly expressed in the stromal myofibroblastic cell line WPMY-1 but weakly in the epithelial cell line RWPE1

**Figure 6:**
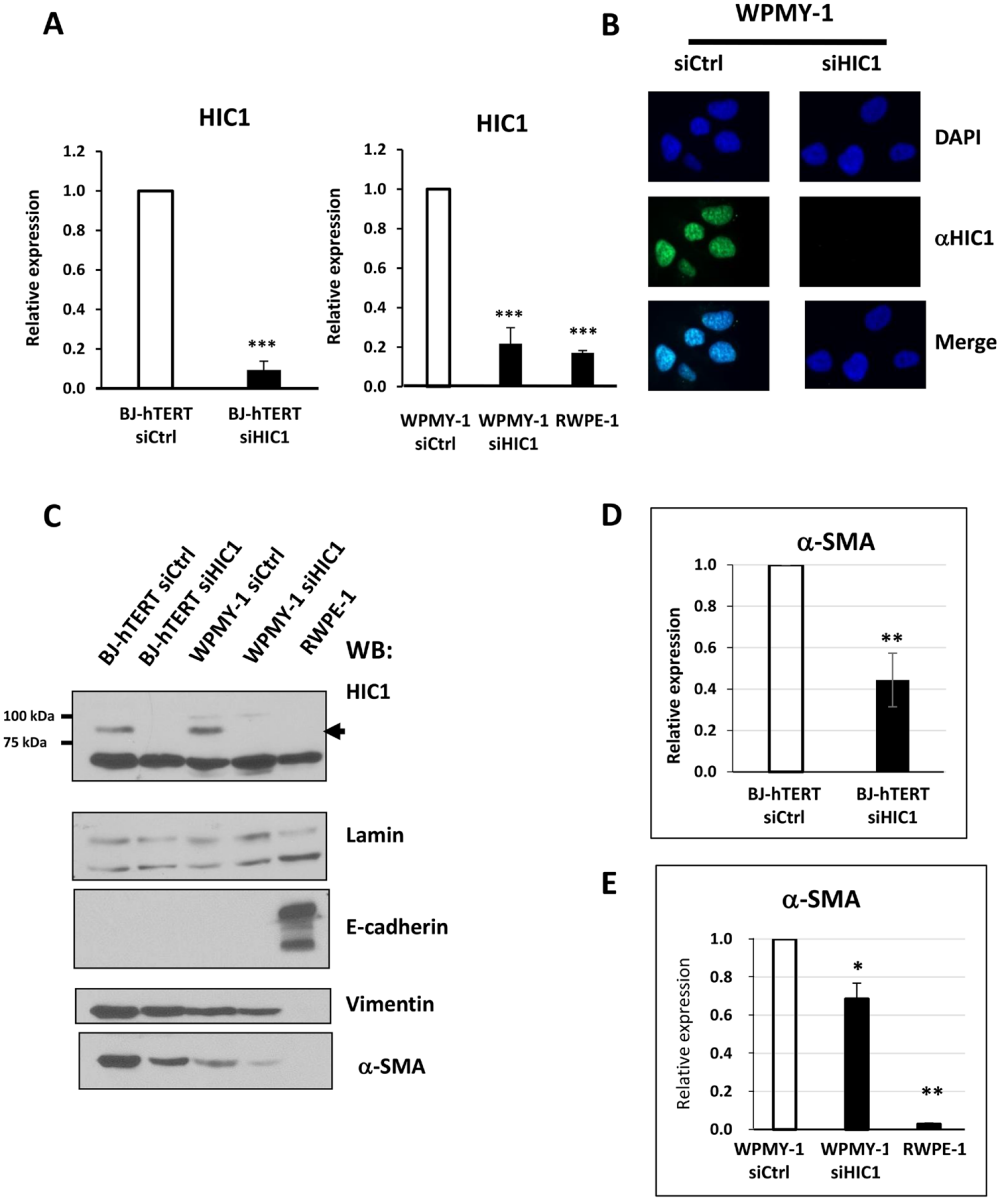
Analyses of *HIC1* expression in the myofibroblastic cell line WPMY-1 compared to the epithelial cell line RWPE1. (**A**) The global expression level of *HIC1* expression was examined by RT-qPCR analyses in two normal immortalized cell lines derived from the same histologically normal prostate [27]: the prostate epithelial cell line, RWPE1 and the stromal myofibroblastic cell line WPMY-1. BJ-hTERT fibroblasts, used as control, and WPMY-1 cells were transfected with the indicated siRNAs. RNAs and total cell extracts (used in Panels C and D) were simultaneously prepared from these cells. (**B**) Immunofluorescence analyses of WPMY-1 cells. WPMY-1 cells transfected with the indicated siRNAs were fixed with paraformaldehyde and analyzed by conventional immunofluorescence microscopy using the monoclonal anti-HIC1 antibody (H-6). Nuclei are seen as DAPI-positive staining. The merging of the two images is shown in the two bottom panels. (**C**) Detection of endogenous *HIC1* proteins by Western blot analyses. Total cell extracts were immunoblotted with the monoclonal anti-HIC1 (H-6) antibodies (upper panels). Immunoblotting with anti-Lamin A antibodies was used as a loading control. E-cadherin and Vimentin or α-SMA were used as markers of epithelial and fibroblastic cells, respectively. (**D** and **E**) *α-SMA* expression is reduced upon *HIC1* knockdown in BJ-hTERT fibroblasts and in WPMY-1 fibroblasts, respectively. *α-SMA* mRNA levels were analyzed by RT-qPCR in the RNA samples used in panel (A) for *HIC1*.

In order to further confirm *HIC1* expression in the two major types of prostatic lineages and to gain access to suitable models for *in vitro* studies, we tested two normal immortalized human cell lines which were derived from the same histologically normal prostate from a 54-year old patient: the widely used RWPE1 epithelial cell line and WPMY-1, a cell line determined to be myofibroblastic on the basis of co-expression of smooth muscle alpha-actin (*a-*SMA) and vimentin [27]. We first confirmed *HIC1* expression by RT-qPCR in WPMY-1 myofibroblasts transfected with a control siRNA (Figure 6A). By contrast, *HIC1* was detected at much lower levels in the RWPE1 epithelial cell line as already observed in primary epithelial cells, PrEC (Figure 4) and similar to the residual levels observed in WPMY-1 myofibroblasts after *HIC1* specific knockdown (Figure 6A). These results were further validated at the protein level by two independent detection methods. First by immunofluorescence microscopy in WPMY-1, endogenous HIC1 proteins were detected by monoclonal anti-HIC1 antibodies in punctate nuclear structures, as already described for numerous proteins with a BTB/POZ domain [28, 29]. As control of antibody specificity, these HIC1-positive nuclear dots were not detectable anymore in WPMY-1 cells transfected with a specific anti-HIC1 siRNA and in WPMY-1 cells incubated with secondary antibodies only (Supplementary Figure 1). We next performed immuno-stainings using total protein extracts prepared from RWPE1, BJ-TERT and WPMY-1 cells transfected with control or HIC1-specific siRNAs [25]. Again, endogenous HIC1 proteins were detected in BJ-hTERT and WPMY-1 cells treated with control siRNA but in neither of these cells after *HIC1* knockdown or in the RWPE1 epithelial cells (Figure 6C). Thus, HIC1 is strongly expressed in fibroblasts and in myofibroblasts.

### *HIC1* inactivation reduces the contractile capability of WPMY-1 cells

In the experiments described above, we used detection of E-Cadherin and Vimentin or alpha-Smooth Muscle Actin (α-SMA) as markers for epithelial and fibroblastic cell identity, respectively. Strikingly, we noticed that the endogenous levels of α-SMA proteins were significantly decreased both in BJ-hTERT fibroblasts and WPMY-1 myofibroblasts upon *HIC1* knockdown (Figure 6C, lower panel). RT-qPCR studies confirmed that depletion of the transcriptional repressor *HIC1* induced a reduction in *α*-SMA at the gene expression level, notably in BJ-hTERT fibroblasts with two-fold decreases observed (Figure 6D and 6E).

**Figure 7:**
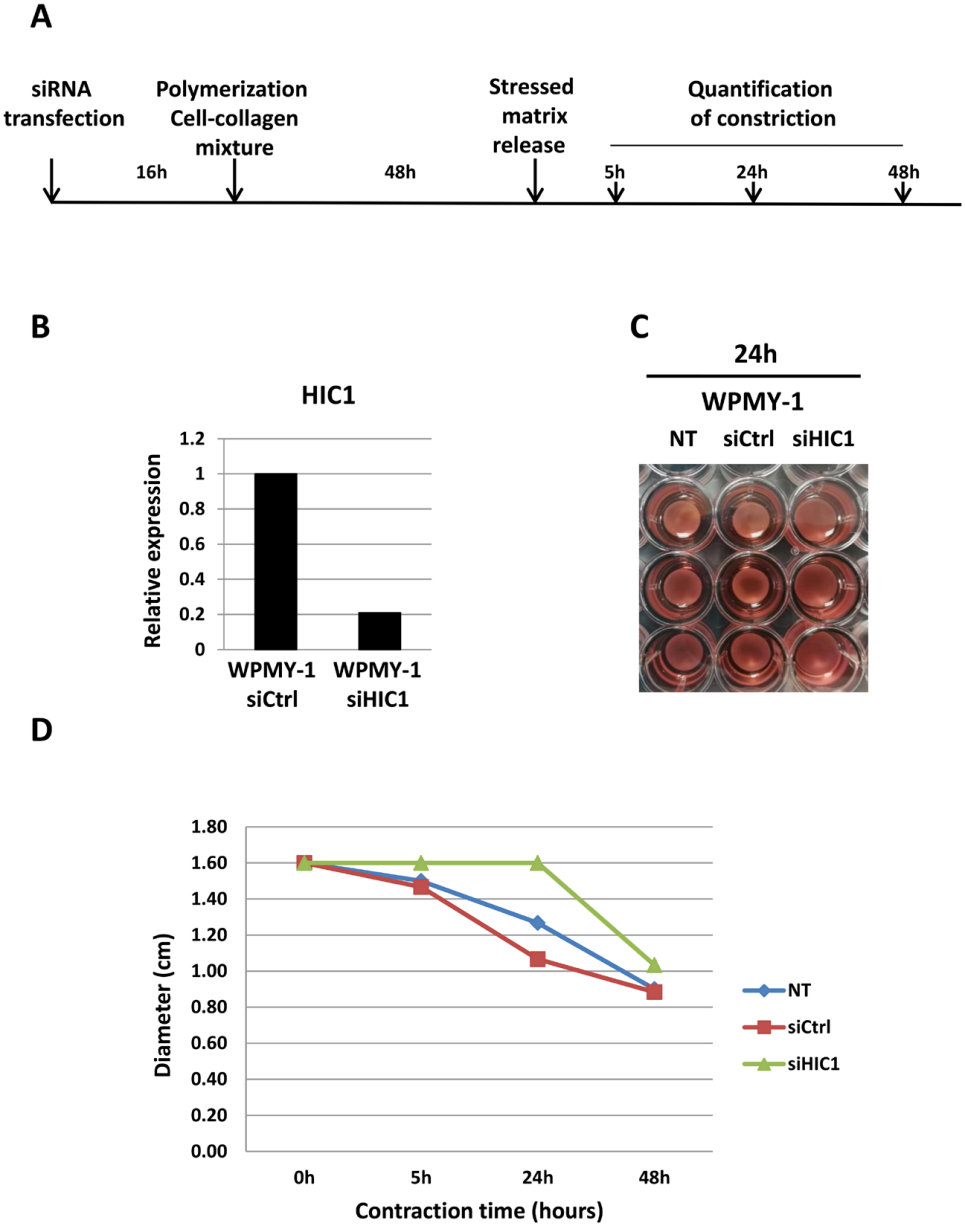
*HIC1* inactivation impairs the contractility of the myofibroblastic cell line WPMY-1. (**A**) Schematic protocol for the contraction assay (detailed in the Material and Methods Section). (**B**) The efficiency of *HIC1* inactivation was checked by RT-qPCR analyses of an aliquot of transfected cells not mixed with collagen but grown in normal medium for 48 hours. (**C** and **D**) Comparison of the contractile capability of non-transfected, siCtrl- and siHIC1- transfected WPMY-1 cells. Quantification of the % of contraction relative to the initial size of the gel area has been performed at the indicated times after stressed matrix release using ImageJ. Representative images obtained after 24 hours are shown in panel (C).

Besides its role as a marker of myofibroblast differentiation, α-SMA expression is correlated with fibroblast contractile activity [30]. We, thus decided to measure the impact the of *HIC1* inactivation by siRNA on the ability of WPMY-1 myofibroblasts to contract using a collagen-based cell contraction assay (Figure 7A). These experiments clearly demonstrated that cell contraction was severely impaired in WPMY-1 myofibroblasts upon *HIC1* specific knockdown as compared to non-transfected cells or cells transfected with control siRNA (Figure 7B–7D). Thus, the contractility of *HIC1*-depleted WPMY-1 cells is decreased concomitant with the reduced expression of α-SMA.

**Figure 8:**
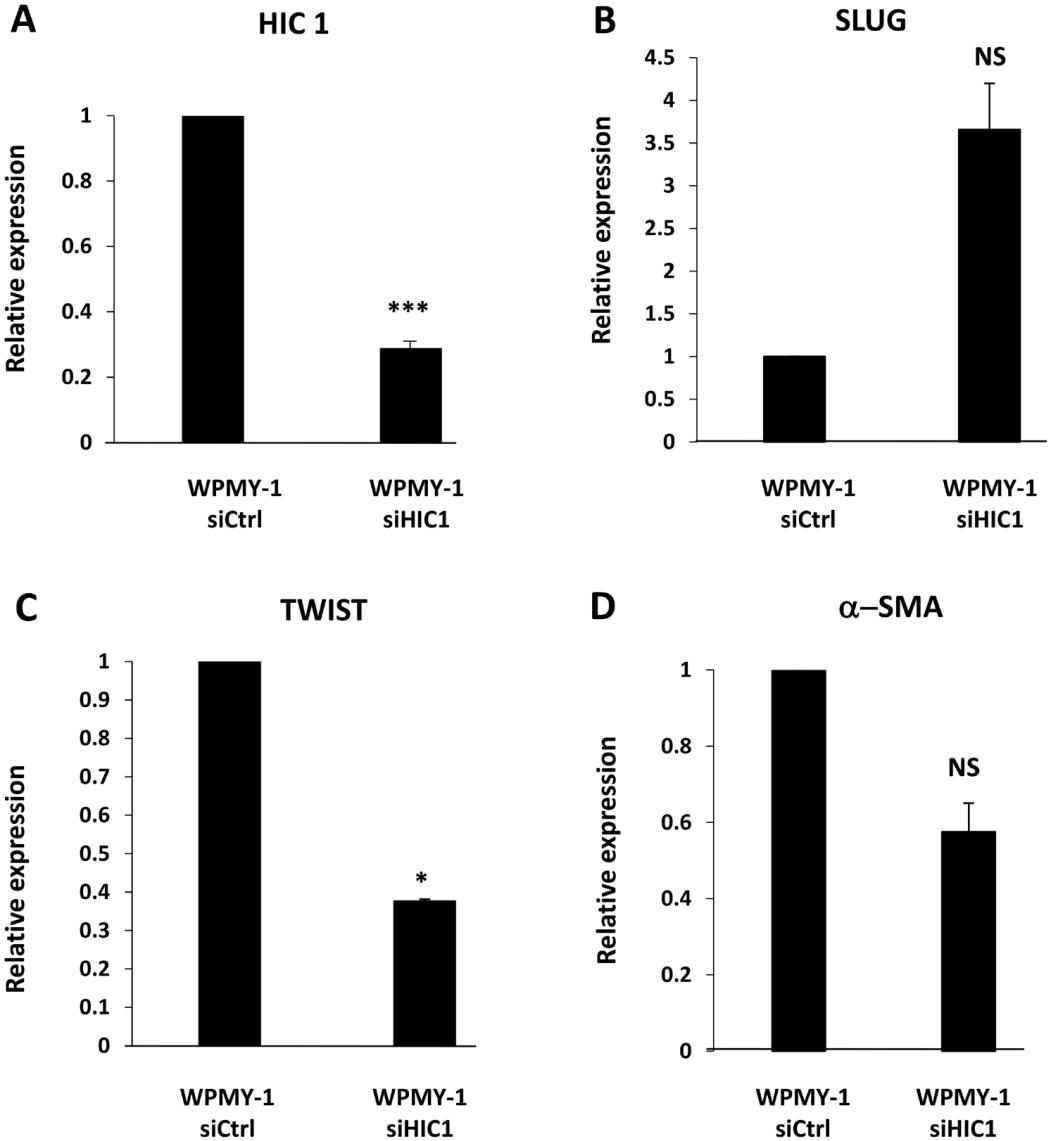
*HIC1* regulates the expression of the EMT-inducing transcription factor *SLUG* in the myofibroblastic cell line WPMY-1. (**A**) The stromal myofibroblastic cell line WPMY-1 was transfected with the indicated siRNAs. RNAs were prepared from these cells and the global expression level of *HIC1* expression was first checked by RT-qPCR analyses. (**B**) The global expression level of *SLUG* was examined by RT-qPCR analyses upon *HIC1* knockdown in the stromal myofibroblastic cell line WPMY-1 cells. (**C**) A similar RT-qPCR experiment was performed on the same samples with the other EMT-inducing transcription factor, TWIST. (**D**) α*-SMA* expression was reduced upon *HIC1* knockdown in WPMY-1 myofibroblasts. As control, α*-SMA* mRNA levels were analyzed by RT-qPCR in the RNA samples used in panel (A–C).

In addition, *α*-SMA is also a marker for EMT (epithelial-mesenchymal transition) associated with tissue fibrosis or invasion and metastasis of cancer cells [31]. In prostate cancer cells, HIC1 directly represses the expression of *SLUG*, an EMT-inducing transcription factor [16]. In WPMY-1 cells with *HIC1* knock-down, RT-qPCR studies confirmed that the expression of *SLUG* was also up-regulated confirming that HIC1 is important for the regulation of *SLUG* both in epithelial [16] and stromal cells (Figure 8B). By contrast, TWIST, another EMT-inducing transcription factor was down-regulated upon *HIC1* knock-down (Figure 8C).

Thus, *HIC1* is strongly expressed in the myofibroblast prostatic cell line, WPMY-1 and is associated with maintained expression of the cell identity marker α-SMA, contractile ability and regulation of the EMT-inducing transcription factor, SLUG.

### Identification of *CXCL12/SDF1* as a new direct target gene of *HIC1* in the prostate stromal myofibroblastic immortalized cell line WPMY-1

**Figure 9:**
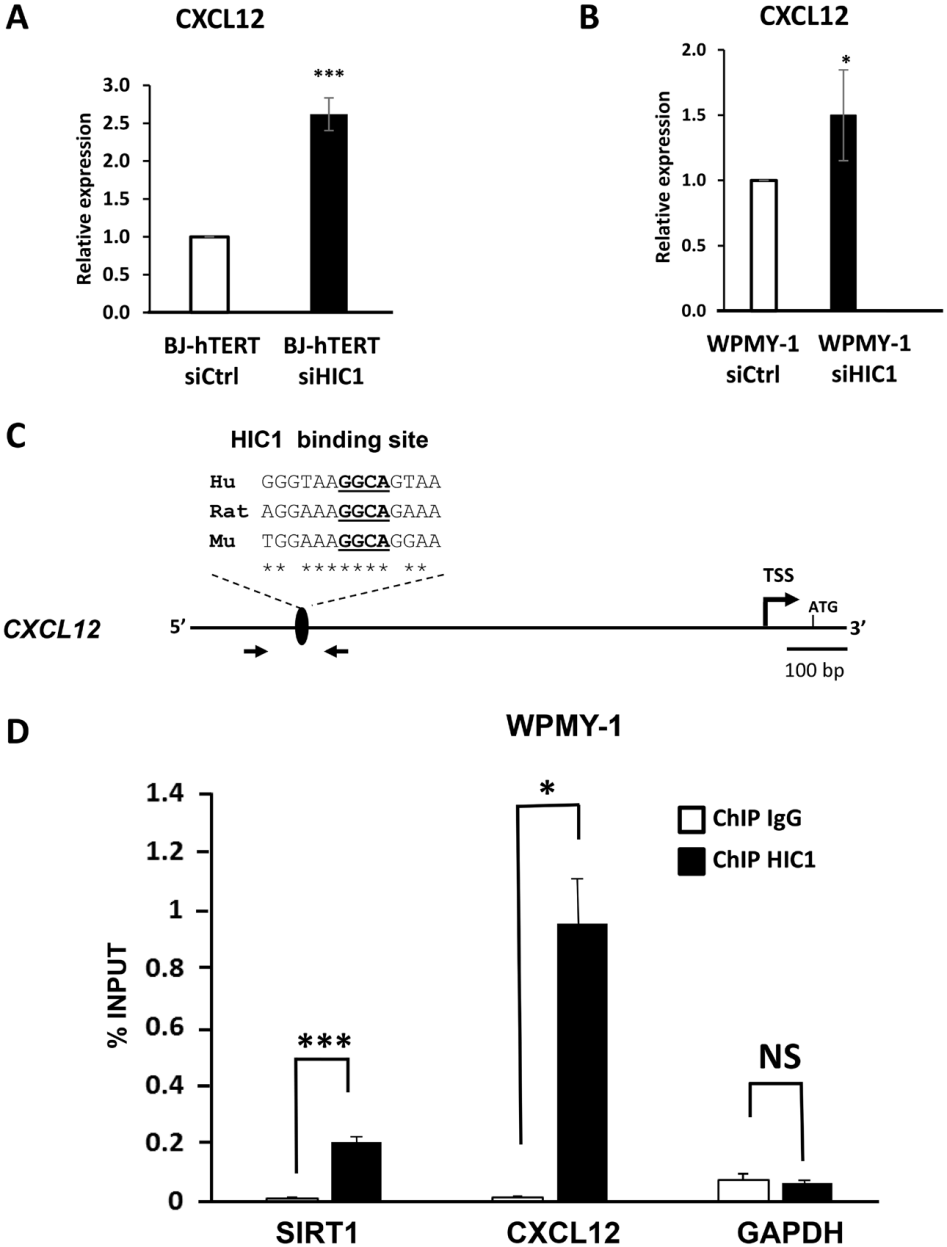
*CXCL12/SDF1* is a direct target gene of HIC1 in the myofibroblastic cell line WPMY-1. (**A** and **B**) The global expression level of *CXCL12* was examined by RT-qPCR analyses after *HIC1* knockdown (RNAs samples used in Figure 5) in two normal immortalized fibroblastic cell lines: the BJ-hTERT foreskin fibroblasts and the stromal myofibroblastic cell line WPMY-1. (**C**) Schematic drawing of the human *CXCL12* proximal promoter region. The transcription start site (TSS) [34], the phylogenetically conserved HIC1 binding site identified through CLUSTAL analyses with the core GGCA motif underlined [19] and the oligonucleotides used in the ChIP assay are shown. Hu: Human; Mu murine, Rat: Rat. (**D**) HIC1 binds the *CXCL12* promoter. Chromatin was prepared from WPMY-1 cells and ChIP analyses were performed for HIC1 or IgG at a conserved HIC1 binding site in *CXCL12* promoter. SIRT1 and GAPDH were used as a positive and non-binding controls, respectively. Values that are statistically different are indicated by bars and asterisks as follows: ^*^ *P* < 0.05 and ^***^ *P* < 0.001. Values that are not statistically significantly different are also indicated (NS).

Microarray analyses comparing BJ-hTERT fibroblasts transfected with a control siRNA or siRNAs targeting *HIC1* identified the stromal derived factor *CXCL12/SDF1* among the up-regulated genes [32]. In addition, *SDF1* is strongly repressed in MCF7 cells overexpressing *HIC1* through infection by an adenoviral vector [33]. These results together with the high expression levels of *HIC1* in stromal cells (Figures 2 and 4) prompted us to investigate the potential direct regulation of the stromal derived factor (*CXCL12/SDF1)* expression by HIC1. RT-qPCR analyses of RNAs prepared from BJ-hTERT and WPMY-1 treated with specific siRNAs targeting *HIC1* (Figure 6A) demonstrated that *CXCL12* expression was up-regulated in both cell types upon *HIC1* inactivation (Figure 9A and 9B). To determine whether *CXCL12* is indeed a direct target gene of HIC1, we performed chromatin immunoprecipitation (ChIP) assays. We designed oligonucleotides flanking a putative HIC1 binding site (HiRE) [19] conserved in the human, rat and mouse genome with the core GGCA HIC1 binding motif localized at position -871/-868 relative to the main *CXCL12* transcriptional start site [34] (Figure 9C). ChIP-qPCR assays using these primers clearly demonstrated that this region of the *CXCL12* promoter was specifically enriched in HIC1-immunoprecipitated chromatin from WPMY-1 as compared to chromatin immunoprecipitated by control mouse IgG (Figure 9D). *SIRT1* and *GAPDH* promoters were included as positive and negative controls, respectively. Similar results were obtained in BJ-hTERT fibroblasts (Supplementary Figure 2). Taken together, these results identified *CXCL12/SDF1* as a new HIC1 direct target-gene in BJ-hTERT fibroblasts and WPMY-1 myofibroblasts.

## DISCUSSION

*HIC1* is a tumor suppressor gene frequently inactivated by promoter hypermethylation in many types of human cancers. In this study, we analyzed *HIC1* expression at protein level by immunohistochemistry (IHC) in normal and prostate tumor tissues of various grade, and at RNA expression level by RT-qPCR in primary prostate tumors as well as in various primary, immortalized or transformed epithelial and stromal prostatic cells. These experiments showed that HIC1 is barely detectable at the protein and RNA levels in prostatic epithelial cells regardless of their transformation status but is strongly expressed in normal and immortalized stromal myofibroblastic cells. Inactivation of HIC1 in WPMY-1 greatly reduced their contractile capability in agreement with the reduced expression of α-SMA. Another interesting finding was that HIC1 directly repressed the expression of *CXCL12/SDF1*, a key player in the complex stromal-epithelial interactions regulating epithelial cell growth. Perturbation of this crosstalk in aging prostate could lead to benign hyperplasia and ultimately to tumorigenesis.

For the analyses of normal prostate and prostate tumors, we used an antibody (Abcam ab33029) recommended for IHC studies that we also validated for the detection of endogenous HIC1 proteins by immunoblotting (Figure 2A). Using this antibody, we showed that HIC1 is undetectable in epithelial cells but rather is detected in the normal and tumor stroma (Figure 2B and 2C). Thus, our results are in contradiction with previous reports using another HIC1 antibody in human prostate tumors [15] or in a murine prostate cancer model with conditional knock-out alleles of *Pten* and/or *Hic1* in prostate [16]. By contrast, our results are in strong agreement with a study addressing the functional role for 3′ CpG island methylation in developmental gene regulation and analyzing the expression of *HIC1* in embryonic (E18.5) mouse colon by RT-qPCR and IHC analyses with the same ab33029 antibody [35]. Using a mouse model of LGR5-eGFP reporter knock-in mice and cell sorting with GFP and EpCAM (a pan-epithelial differentiation antigen), these authors isolated different cell subpopulations from colonic mucosa and demonstrated that *HIC1* is exclusively mesenchymal. Indeed, *HIC1* expression is very low in colonic epithelial stem cells (Lgr5^-^/eGFP^+^) and in differentiated epithelial cells (EpCAM^+^) but very robust in the remainder of mesenchymal cells (EpCAM^-^/Lgr5^-^/eGFP^-^). IHC with the ab33029 anti-HIC1 antibody we used, confirmed that HIC1 is exclusively mesenchymal and displayed a strong expression at the outer layer of the *mucosa externa* in cells also labeled with the mesenchyme specific marker, α-SMA [35]. Finally, in normal human colon, HIC1 is detected by IHC with the same ab33029 antibody in the epithelium and in the *lamina propria*, which is located beneath the intestinal crypts and contains various mesenchymal cells including fibroblasts and myofibroblasts [10, 36]. HIC1 is also detected in the stroma of both high- and low-HIC1 expressing colon carcinomas (CRCs) and in the epithelial cancer cells of high-HIC1 CRCs which principally belong to the CMS4 subtype characterized by the worst survival and a strong stromal gene signature [10]. In conclusion, the detection of HIC1 by IHC appears to be technically challenging, resulting in conflicting results that might be attributed to the use of different antibodies and/or protocols and also to still poorly understood tissue-, age- or gender- specific regulations of *HIC1* expression [18].

To overcome these conflicting observations and to bolster our immunohistochemistry data, we measured *HIC1* RNA expression levels by RT-qPCR in various tissues and cellular models and we took advantage of a recently published single cell RNA-sequencing dataset in normal prostate (Figure 5). Firstly, analyses of 30 prostate tumors strongly suggested that *HIC1* expression is correlated with tumor stroma content (Figure 3), as previously shown for colon carcinomas [10]. Secondly, *HIC1* expression was not detected in three transformed epithelial cell lines tested in close agreement with the silencing of *HIC1* through promoter hypermethylation in many cell lines [6] and tumors, including prostate tumors [12–16]. Finally, *HIC1* expression was barely detected in primary prostate epithelial cells (PrEC) but detected in primary smooth muscle cells (PrSMC) (Figure 4) in strong agreement with our IHC data and with bulk RNA-Seq analyses of isolated cell populations from human prostate (Figure 5; data retrieved from reference 26). In another study, *HIC1* expression was detected in PrEC by semi-quantitative gel-based assays and Western blot analyses [15]. This discrepancy could be due to genetic drift of PrEC, to different culture conditions and/or to the differences in the detection assays used (e.g., semi-quantitative versus quantitative RT-PCR and different antibodies). *HIC1* promoter is not methylated in PrEC [15]. Nevertheless, the unmethylated state of the *HIC1* promoter in PrEC is not necessarily coupled to strong transcriptional activity. In fact, inactivation of *HIC1* is not always due to promoter hypermethylation as shown for example in acute myeloid leukemias [37].

Finally, in line with our results, we detected a strong expression of *HIC1* in WPMY-1 myofibroblasts as compared to a low basal expression in RWPE1 epithelial cells; two immortalized cell lines derived from the same prostate [27]. During wound healing, the release of cytokines and growth factors by inflammatory infiltrating immune cells leads to the activation and trans-differentiation of quiescent fibroblasts into contractile myofibroblasts allowing the wound to close. These activated fibroblasts express α-smooth muscle actin (α-SMA), hence the name “myofibroblast”. In tumors, often considered to be “wounds that do not heal”, myofibroblasts are designated as Cancer-Associated Fibroblasts (CAFs), which play a key role in tumor growth and progression [38]. We observed that α-SMA expression was significantly decreased in WMPY-1 myofibroblasts with inactivated *HIC1* (Figure 6). We demonstrated, using a functional contraction assay, that *HIC1* inactivation diminished the contractile activity of WPMY-1 cells (Figure 7). Recently, in a conditional knock-out model of murine *Hic1*, the contractility of seminiferous tubules was also shown to be reduced due to the decreased expression of the smooth muscle contractile proteins α-SMA (Acta2) and Calponin1 (Cnn1) [39]. In addition, a similar link between HIC1 and α-SMA has been demonstrated in a human model of chronic wound healing, namely normal human dermal fibroblasts (NHDF) treated with TGF-β or with filtered exudates from chronic wounds. Five transcription factors (TFs), including HIC1, were defined as essential for fibroblast to myofibroblast differentiation monitored by analyzing α-SMA expression. Indeed, individual siRNA knockdown of these TFs in NHDF fully inhibited the expression of α-SMA as compared to its expression in NHDF treated with TGF-β [40]. Finally, Janeckova and coworkers noticed a significant overlap between the genes up-regulated in the HIC1-high CRC subtype they identified and the TGF-β response gene signatures in different types of stromal cells [10, 41] and concluded that, at least in some CRCs, *HIC1* is expressed in the stroma rather than in epithelial tumor cells. Through data mining, they also found evidence for increased expression of *HIC1* in primary lung cancer cells co-cultured with CAFs as well as for an increased expression of *HIC1* in non-small cell lung cancer cells treated with TGF-β [10]. As a whole, these data suggest the existence of an intricate relationship between the HIC1 and TGF-β pathways in stromal and cancer cells that deserves to be further investigated.

Finally, our work reveals a new role for HIC1 in the regulation of the CXCL12/CXCR4 axis. This chemokine receptor-ligand signaling pathway is implicated in the growth, invasion and metastatic properties of many cancer cells, including prostate cancer [3–5, 42]. Interestingly, *CXCR4*, the *bona fide* CXCL12 receptor and *CXCR7*, a scavenger receptor overexpressed in prostate cancers, have been both identified as direct target genes of HIC1 [16, 43, 44]. Previously, we noticed that *CXCL12/SDF1* was up-regulated in microarray analyses comparing BJ-hTERT fibroblasts transfected with a control siRNA or siRNAs targeting *HIC1* [32]. Furthermore, *SDF1* appears strongly repressed in MCF7 cells overexpressing *HIC1* through infection by an adenoviral vector [33]. Here, using RT-qPCR and ChIP assays, we demonstrated that *CXCL12/SDF1* is in fact a direct target gene of HIC1 in WMPY-1 stromal cells. In a recent study, its receptor, CXCR4, which is expressed at the membrane of epithelial cells, and Slug, a transcription factor known to induce Epithelial to Mesenchyme Transition (EMT) were both shown to be direct HIC1 target genes, using classical ectopic overexpression models of HIC1 in transformed prostate cell lines [16]. These authors thus concluded that impairment of *HIC1* expression in prostate cancer cells induces their migration and metastasis by promoting EMT at two levels through increased expression of the transcription factor SLUG and of the CXCR4 receptor. Here, we demonstrated that SLUG expression is also up-regulated in WPMY-1 stromal cells upon *HIC1* knock-down (Figure 8). Furthermore, SLUG can promote prostate cancer cell migration and invasion via activation of the CXCR4/CXCL12 axis [45]. Indeed, the CXCL12/CXCR4 axis is known to induce EMT through activation of the Erk1/2 pathway [46]. *HIC1* silencing in tumors could thus induce a synergistic activation of the paracrine CXCL12/CXCR4 pathway by simultaneous direct transcriptional activation and indirect activation mediated by SLUG of the CXCL12/SDF1 ligand, which is secreted by stromal cells and of its receptor located on epithelial cells. This mechanism could also be implicated in early prostate tumorigenesis. Indeed, prostate cancer incidence increases with age [2] and *HIC1* is epigenetically silenced in normal prostate aging [12]. The levels of CXCL12 chemokine found in conditioned medium (CM) of stromal fibroblasts isolated from the prostate of men 63-81 years-old at the time of surgery is higher when compared with those in the CM of cells isolated from younger men [46]. This relies on a strong up-regulation of *CXCL12* expression as shown by gene profiling experiments comparing fibroblast cell populations from 4 younger and 2 older patients [46].

In conclusion, our results demonstrate that *HIC1* is expressed in stromal cells and directly regulates the expression of *SDF1/CXCL12*. These studies provide new insights into the role of HIC1 in normal prostatic epithelial-stromal interactions and new mechanistic clues on how its loss of function through promoter hypermethylation during aging could increase epithelial cell proliferation, thereby contributing to benign hyperplasia and ultimately to prostatic tumors.

## MATERIALS AND METHODS

### Patient information and tissue selection

All PCa patients included in this study had undergone radical prostatectomy in the Lille University Hospital. Clinical data and patient consent were obtained by the referring physician (Supplementary Table 1). Total RNAs used in RT-qPCR analyses were extracted from frozen tissues corresponding to matched healthy prostate and neoplastic tissue obtained from 10 patients and to primary tumors obtained from 20 other patients (age ranging from 51 to 73 years; Gleason score ranging from 6 to 9; see Supplementary Table 1 for details) as previously described [24, 47]. These tissues were obtained from the urological collection of the local tumor tissue bank (Tumorothèque C2RC, CHU LILLE, France) after approval by the internal review board (CSTMT 225). Most of this cohort (Tumors 1–25) has been used in a previous study focusing on the expression of *hPCL3S* [24]. As a control, total prostate RNAs from a healthy 24-year donor were obtained from Biochain.

### Cell lines

PrECs and PrSMCs were obtained from Lonza and grown in complete medium as recommended by the supplier. The WPMY-1 cell line was obtained from the American Type Culture Collection (ATCC). RWPE1, PC3, DU145, LNCaP [48, 49] and BJ-hTERT cells [25] were maintained in Dulbecco modified Eagle medium (Invitrogen) supplemented with 10% fetal calf serum, non-essential amino acids and gentamycin. Cells were cultured at 37°C in humidified 5% CO2 atmosphere as previously described [25, 50].

### Small interfering RNAs

HEK293T cells were reverse-transfected with Lipofectamine RNAiMax (Invitrogen) according to manufacturer’s instructions using 10 nM small interfering RNA targeting HIC1 (Human, Dharmacon), or a scrambled control sequence (si Ctrl; siGENOME RISC free control siRNA, Dharmacon). 48 hours after siRNA transfection, cells were treated for RNA extraction [25].

### Cell contraction assay

Cell contractile capability in WPMY-1 and in WPMY-1 transfected with siRNAs targeting HIC1 or siCtrl was compared using a collagen gel-based cell contraction assay kit (CBA-201; Cell Biolabs Inc, San Diego, CA). After transfection, cells were trypsinized and resuspended in the culture medium at a density of 6 × 10^6^ cells/mL. The collagen lattice was prepared by mixing the cells and the cold Collagen gel working solution at a ratio of 1:4. 0.5 mL of this mixture was added to a well of a 24-well plate and incubated for 1 h at 37°C. After collagen polymerization, 1 mL of culture medium is added on each collagen gel lattice. The cells were incubated for 48 h. To initiate cell contraction, the collagen gel was released with a sterile spatula. Changes in collagen gel size were imaged and quantified using ImageJ.

### RNA isolation and quantitative RT-PCR analyses

Total RNA from the tumor samples and cell lines was purified using the Nucleospin RNA kit (Macherey Nagel) which includes a DNase treatment to eliminate contaminating genomic DNA. Total RNA was reverse transcribed using random primers and MultiScribeTM reverse transcriptase (Applied Biosystems). Real-time PCR analysis was performed by Power SYBR Green (Applied Biosystems) in a MX3005P fluorescence temperature cycler (Agilent) according to the manufacturer’s instructions. The primers used for the RT-qPCR analyses at a concentration of 10.0 μM are summarized in Supplementary Table 2. According to a melting point analysis, only one PCR product was amplified under these conditions. In all experiments, a “no-RT” control was included to exclude contamination by genomic DNA. Results were normalized with respect to *ALAS1* [51] or *18S* RNA used as an internal control.

### Statistics

Experiments were performed at least twice in duplicate or triplicate. Statistical analyses were performed by Student’s test. (^*^) indicates *p* < 0.05, (^**^) indicates *p* < 0.005 and (^***^) indicates *p* < 0.001. NS: Non-Significant.

### Immunohistochemistry

To evaluate HIC1 protein expression, immunohistochemistry was performed as previously described [47–49] using anti-HIC1 antibodies (ab33029, Abcam, Cambridge, UK). Immunohistochemistry was performed using an automated immunostainer (Benchmark XT, Ventana). Briefly, after deparaffinization and pretreatment in a Tris-base buffer CC1, pH8.4 during 76’, the primary antibody was incubated for 45 min, dilution 1/50, at 37°C. An amplification was performed using Ultraview kit (Amplifia Ultraview, Ventana).

### Antibodies, Western blot and Immunofluorescence analyses

Commercial primary antibodies of the following specificities were used: HIC1 (mouse monoclonal antibody against HIC1 C-terminal Amino Acids 611-733: H-6 Santa Cruz, sc-271499, dilution 1/200), Lamin (Santa Cruz, sc-20681), E-cadherin (Santa Cruz, sc-7870), Vimentin (Santa Cruz, sc-6260) and α-SMA (ab5694, Abcam). Western blots were performed as previously described [25]. The secondary antibodies were horse radish peroxidase-linked antibodies against rabbit and mouse immunoglobulins (Amersham Biosciences) or goat immunoglobulins (Southern Biotech). Immunofluorescence analyses were performed essentially as previously described [52] on WPMY-1 cultured on coverslips using anti-HIC1 H-6 (dilution 1/50) and Alexa Fluor 488 goat anti-mouse IgG (Life Technologies) as secondary antibodies.

### Chromatin immunoprecipitation

WPMY-1 cells were washed with PBS and resuspended in 0.5 ml PBS for 5 × 10^6^ cells. Then, cells were fixed by adding formaldehyde to a final concentration of 1% for 8 min at room temperature. To stop fixation, glycine was added to a final concentration of 0.125M. After 5 min at room temperature, cells were collected by centrifugation (1500 rpm, at 4°C, 5 min). The supernatants were removed and we lysed cells by resuspension in chilled cell lysis buffer for 10 min on a rotator at 4°C. Then, the samples were pelleted, resuspended in 200 μl nuclei lysis buffer and sonicated to chromatin with an average size of 250 bp using a cooling BioRuptor (Diagenode, Belgium). 20 μg of chromatin was immunoprecipitated with indicated antibodies (anti-HIC1 H-6, sc-271499 or normal mouse IgG, sc-2025) and real-time PCR analyses were performed as described [53]. The primers used to amplify the *SDF-1/CXCL12* promoter region were as follows: sense 5′-GGGTCGCACGGAGCTTTT-3′; antisense 5′-TCTGACAAGCAGACAGTACTCAA-3′. The primers used for *GAPDH* and *SIRT1* have been previously described [54].

### Additional resources

To aid our analysis, we used a publicly available gene expression dataset obtained by bulk RNA-sequencing of FACS-sorted cells from normal human prostate tissue (GSE120716) [26]. Single cell RNA-sequencing of human prostate cells was retrieved from Dr Douglas Strand’s laboratory interactive website (https://www.strandlab.net/).

## SUPPLEMENTARY MATERIALS




