# Supplementary material for: HIC1 (Hypermethylated in Cancer 1) modulates the contractile activity of prostate stromal fibroblasts and directly regulates CXCL12 expression
Source: Oncotarget. 2020 Nov 10;11(45):4138–54. doi: 10.18632/oncotarget.27786 (PMC7665237; doi:10.18632/oncotarget.27786)
Supplement: Supplementary file 1 [file oncotarget-11-4138-s001.pdf]

HIC1 (Hypermethylated in Cancer 1) modulates the contractile activity of prostate stromal fibroblasts and directly regulates *CXCL12* expression

SUPPLEMENTARY MATERIALS

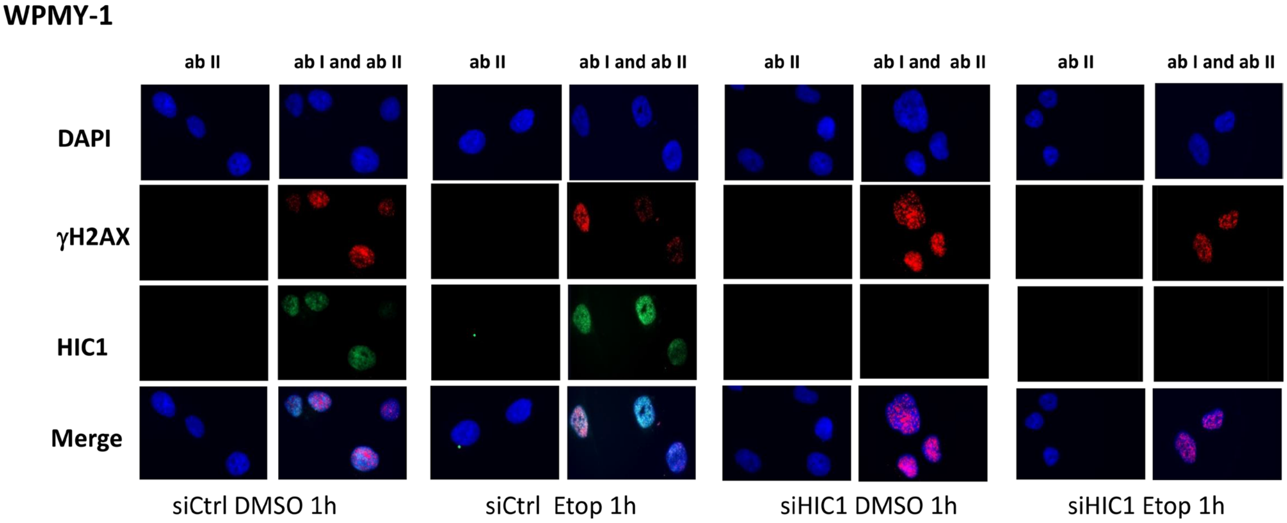

Supplementary Figure 1: Control immunofluorescence analyses of WPMY-1 cells. WPMY-1 cells transfected with the indicated siRNAs were treated for 1 hour with Etoposide (20 mM) or with DMSO as control and fixed with paraformaldehyde. For each conditions cells were analyzed by conventional immunofluorescence microscopy using as primary antibody (ab I) either the monoclonal anti-HIC1 antibody (H-6) or an anti-γH2AX antibody to monitor the induction of DNA damages and the relevant secondary antibodies (ab II) (Right columns: ab I + ab II). As controls, cells were also incubated with the secondary antibody alone (Left columns: ab II). Nuclei are seen as DAPI-positive staining. The merging of the two images is shown in the two bottom panels.

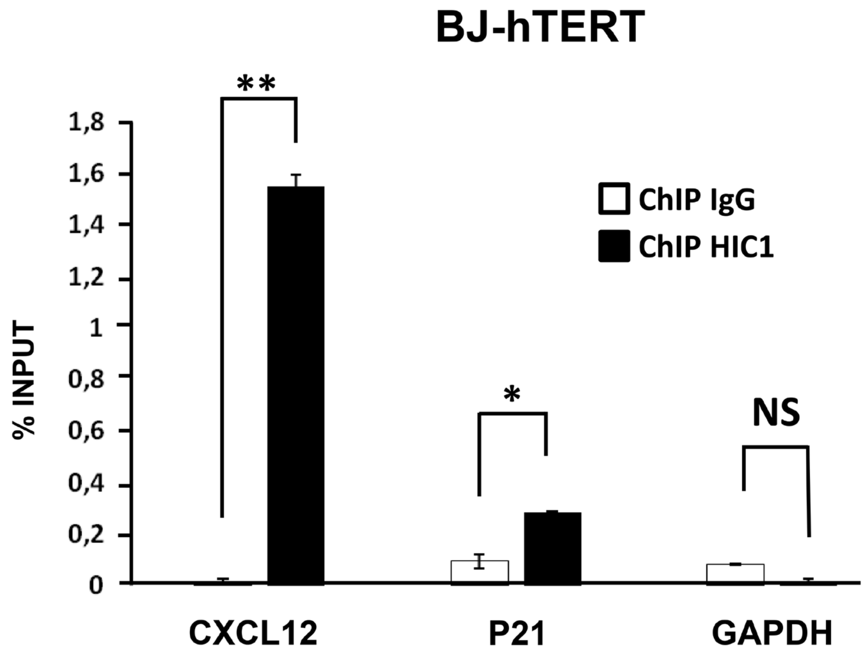

Supplementary Figure 2: HIC1 binds the *CXCL12* promoter. Chromatin was prepared from BJ-hTERT cells and ChIP analyses were performed for HIC1 or IgG at a conserved HIC1 binding site in *CXCL12* promoter. P21 and GAPDH were used as a positive and nonbinding control, respectively. Values that are statistically different are indicated by bars and asterisks as follows: $^{*}P < 0.05$ and $^{***}P < 0.001$. Values that are not statistically significantly different are also indicated (NS).

Supplementary Table 1: Clinical/pathological characteristics of the prostate tumors. See Supplementary Table 1

Supplementary Table 2: Oligos used in the RT-qPCR experiments

RT- qPCR		
**HIC1 Total**	Forward	CGACGACTACAAGAGCAGCAGC
**HIC1 Total**	Reverse	CAGGTTGTCACCGAAGCTCTC
**HIC1 Variant 1**	Forward	GACCAGCAGGACAGACCGA
**HIC1 Variant 1**	Reverse	TGGGGGGCATGTCGAAAG
**CXCL12**	Forward	AACGCCAAGGTCGTGGTC
**CXCL12**	Reverse	GCATGGGCATCTGTAGCTCA
**GAPDH**	Forward	GCACCGTCAAGGCTGAGAA
**GAPDH**	Reverse	CGCCCCACTTGATTTTGGA
**TWIST**	Forward	GCCTTCTCGGTCTGGAGGAT
**TWIST**	Reverse	TTCTCTGGAAACAATGACATCTAGG
**SLUG**	Forward	AAGGCGTTTTCCAGACCCTG
**SLUG**	Reverse	AAGAAAAAGGCTTCTCCCCCGT
**α SMA**	Forward	CTGTTTTCCCATCCATTGTG
**α SMA**	Reverse	CCATGTTCTATCGGGTACTT
**18S**	Forward	GGCGCCCCCTCGATGCTCTTAG
**18S**	Reverse	GCTCGGGCCTGCTTTGAACACTCT
**ALAS1**	Forward	TGGTGCAGTAATGACTACCTAGGA
**ALAS1**	Reverse	CCCCAGCACCATGTTGTTT
